# Teaming up to make kombucha

**DOI:** 10.7554/eLife.81670

**Published:** 2022-08-11

**Authors:** Olga Ponomarova

**Affiliations:** 1 Department of Systems Biology at the University of Massachusetts Chan Medical School Worcester United States

**Keywords:** kombucha tea, symbiosis, complexity reduction, fermentation, microbiome, Other

## Abstract

Reducing the microbial diversity in a type of fermented tea reveals the core metabolic interactions responsible for the drink’s signature taste and characteristics.

**Related research article** Huang X, Xin Y, Lu T. 2022. A systematic, complexity-reduction approach to dissect the kombucha tea microbiome. *eLife* **11**:e76401. doi: 10.7554/eLife.76401.

From cheese to salami, to beer or miso soup, chances are that your favorite delicacy owes its unique flavors to humble communities of microorganisms which ferment sugars into substances that preserve and improve food (Bourdichon et al., 2021). Humans have been enthusiastically brewing or pickling since the Bronze Age, yet surprisingly little is known about the intricacies of the fermentation process (Farag et al., 2019; Yang et al., 2014).

Fermenting food requires dozens if not hundreds of microbial species which work closely together, each producing substances which the others take up, use and transform into new chemicals important for other species in the community (Tamang et al., 2016). These complex interactions make it challenging to disentangle how individual actors contribute to the overall process, and to identify the ones essential for the final product. Now, in eLife, Xiaoning Huang, Yongping Xin and Ting Lu report having methodically reduced the complex microbial system which creates the tangy drink known as kombucha tea, down to a single pair of species (Huang et al., 2022).

Kombucha is created by a thriving community of yeast and bacteria which work together to ferment sugary tea. Huang et al. first focused on the features that this culture must have to produce the famous concoction. Three key characteristics emerged: both yeast and bacteria should be present; a characteristic jelly-like film or ‘pellicle’ should form at the surface; and the culture should consume sucrose while accumulating acetate, ethanol, and small amounts of sucrose constituents such as glucose. Preserving these features ensured that a core community of microbes would capture the essential metabolism of the native culture found in kombucha (Figure 1; Step 1).

**Figure 1.**
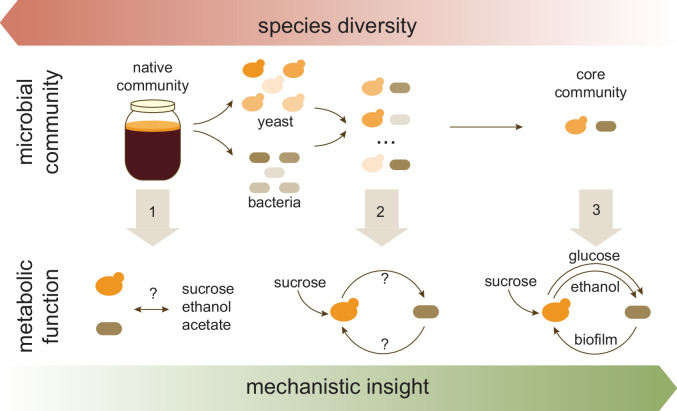
Approach used by Huang et al. to investigate the role of specific members of the microbial community found in kombucha tea. The species richness of the kombucha microbial community was systematically reduced (top), with each step (gray arrows) improving the understanding of the metabolic function of core species in the culture (bottom). Step 1: Analyzing the native kombucha community revealed the identity and relative abundance of its various microbial members; gross metabolic changes were also recorded (such as consumption of sucrose and production of ethanol and acetate), but they were unassigned to any microorganisms. Step 2: Isolating five yeast and five bacterial species and analyzing their twenty-five pairwise combination cultures revealed that the bacteria depended on yeast degrading sucrose. Step 3: In-depth analysis of a representative yeast-bacteria pair revealed the specific interactions underlying their collaboration (cross-feeding of glucose and ethanol from yeast to bacteria, and bacterial production of biofilm which potentially protects the community).

Next, the team (who are based at the University of Illinois Urbana-Champaign and the China Agricultural University) isolated five yeast and five bacterial species, examining each of them individually or as yeast-bacteria pairs. Some bacteria completely depended on yeast to break down sucrose into glucose and into other essential molecules required for their survival (Figure 1; Step 2). Although all yeast species could survive on their own, the distinctive properties of kombucha (such as its pellicle, high acidity and acetate production) occurred only in co-cultures, indicating that bacteria did contribute to these community functions.

To understand how the community worked at an even finer scale, Huang et al. focused on a single yeast-bacteria pair which could create all three features characteristic of native kombucha. This co-culture was remarkably stable: no matter the ratio of yeast to bacteria at the start of the process, the final communities had roughly equal numbers of each species once stable. They also all produced concoctions which closely resembled traditional kombucha, with similar levels of acidity, sugars, ethanol, and acetate.

Next, these two species were individually cultured on diverse nutrient sources to closely monitor which compounds they could consume and produce (Figure 1; Step 3). The manipulation revealed that only the yeast could make glucose and ethanol; this likely involves the cells secreting an enzyme that processes sucrose into glucose, which is then available for ‘public use’ (Tran et al., 2020; Smith and Schuster, 2019). In turn, the bacteria could only create a pellicle when they consumed glucose and ethanol at the same time. This experiment helped to finally piece together how the two species interact: yeast feed and stimulate bacteria with glucose and ethanol, while bacteria wrap the community in a film that may shield it from the environment (Yin et al., 2019).

If two species alone can thrive and produce kombucha-like tea, then why does this process normally involve many more microorganisms? This taxonomic diversity may improve adaptability (Willi et al., 2006), or it may just emerge through random processes (Sloan et al., 2006); it could even be an artefact due to sampling at an inadequately large scale (Fierer and Lennon, 2011). Further studies are needed to investigate these possibilities.

The reductionist approach developed by Huang et al. allows scientists to pinpoint the core subgroups of microbes which perform the primary functions of a wider community, and to disentangle the role of individual species. This framework is useful to understand the metabolic processes responsible for the signature look, taste and smell of fermented foods. The next steps would potentially involve finetuning the method to study microbial communities which are harder to define, such as those that interact with host organisms or the wider environment.
